# Screening and surveillance of esophageal cancer by magnifying endoscopy with narrow band imaging improves the survival of hypopharyngeal cancer patients

**DOI:** 10.3389/fonc.2023.1221616

**Published:** 2024-01-23

**Authors:** Chen-Shuan Chung, Chia-Yun Wu, Yu-Hsuan Lin, Wu-Chia Lo, Ping-Chia Cheng, Wan-Lun Hsu, Li-Jen Liao

**Affiliations:** ^1^ Division of Gastroenterology and Hepatology, Far Eastern Memorial Hospital, New Taipei, Taiwan; ^2^ College of Medicine, Fu Jen Catholic University, New Taipei, Taiwan; ^3^ Department of Oncology and Hematology, Far Eastern Memorial Hospital, New Taipei, Taiwan; ^4^ Head and Neck Cancer Surveillance & Research Group, Far Eastern Memorial Hospital, New Taipei, Taiwan; ^5^ Master’s Program of Big Data Analysis in Biomedicine, Fu Jen Catholic University, New Taipei, Taiwan; ^6^ Department of Otolaryngology, Far Eastern Memorial Hospital, New Taipei, Taiwan; ^7^ Graduate Institute of Medicine, Yuan Ze University, Taoyuan, Taiwan; ^8^ Department of Electrical Engineering, Yuan Ze University, Taoyuan, Taiwan

**Keywords:** narrow band imaging (NBI), screening, esophageal cancer, head and neck (H&N) cancer, second primary tumors (SPTs)

## Abstract

**Introduction:**

Patients with head and neck cancer may develop a second primary neoplasm (SPN) of the esophagus due to field cancerization. This study investigated the impacts of esophageal cancer screening using magnifying endoscopy with narrow-band imaging (ME-NBI) on the outcomes of hypopharyngeal cancer patients.

**Methods:**

Patients with hypopharyngeal cancer diagnosed from 2008 to 2021 in a tertiary hospital were reviewed retrospectively. Screening and surveillance using ME-NBI examination of the esophagus were divided into three patterns: (1) ME-NBI never performed or more than 6 months after diagnosis of index primary hypopharyngeal cancer, (2) ME-NBI within 6 months only, and (3) ME-NBI within 6 months and regular surveillance.

**Results:**

A total of 261 were reviewed and 21 (8%) patients were in stage I, 20 (8%) in stage II, 27 (10%) in stage III, 116 (44%) in stage IVA, 65 (25%) in stage IVB, and 12 (5%) in stage IVC. Sixty-seven (26%) patients had SPN (50 esophagus, 10 oral cavity, 3 oropharynx, 2 nasopharynx, 1 larynx and 1 lung). Among esophageal SPN, 35 (70%) and 15 (30%) patients developed synchronous and metachronous neoplasia, respectively. In multivariate Cox regression analysis, advanced stages III and IV (compared with stages I and II, HR: 1.86, 1.18-2.95, p=0.008), ME-NBI examination of the esophagus received within 6 months and regular surveillance (HR: 0.53, 0.36-0.78, p=0.001) were independent factors affecting the overall survival of patients with hypopharyngeal cancer.

**Discussion:**

Our findings demonstrated that screening and surveillance of esophageal SPN by ME-NBI improves the survival of patients with hypopharyngeal cancer.

## Introduction

Currently, head and neck cancer (HNC) and esophageal cancer are among the top ten causes of cancer death (1, 2). Most of the deaths from HNC are due to disease recurrence and progression. HNC is a malignancy that develops in the oral cavity and pharynx, including the nasopharynx, oropharynx, hypopharynx, and pharynx or larynx. Its occurrence is closely related to carcinogen consumption, such as cigarette smoking, alcohol drinking, betel quid chewing and human papillomavirus exposure (2). Chewing betel quid is part of the culture in some Asian countries, and the incidence rates of HNC are higher in these regions (3). Compared with other HNCs, hypopharyngeal cancer is relatively rare, accounting for approximately 3% of all HNCs (4–6). Unfortunately, hypopharyngeal cancer has the worst prognosis, with a reported 5-year survival rate of approximately 30-35% (4).

Anatomically, the hypopharynx is defined by its subregions, including the posterior hypopharyngeal wall, the lateral pyriform sinus, and the postcricoid area, which is an entrance to the esophagus. Hypopharyngeal cancer often presents at an advanced symptomatic stage and requires aggressive treatment. This disease can greatly affect the patient’s quality of life (5). Despite medical advances in treatment, the overall oncological prognosis of hypopharyngeal cancer remains relatively poor (6). One of the most important factors for this dismal malignancy is the occurrence of a second primary neoplasm (SPN) of the head and neck region, lung and esophagus (7, 8). The prevalence of SPN was approximately 12%, with the most common site being the head and neck region, followed by the lungs and esophagus in a meta-analysis of 51,454 HNC patients, and 13% of them were reported to have high-grade dysplasia or invasive carcinoma of the esophagus (9). A nationwide cohort study of 9,996 SPNs recorded among 93,891 HNC patients demonstrated the worst prognosis with SPNs of the esophagus and lung, with a cure rate of only 11% (10). Additionally, SPNs of the esophagus may occur synchronously and metachronously. The 5- and 10-year cumulative incidence rates of metachronous esophageal cancer have been reported as 1.4% and 2.7%, respectively, among HNC patients with a negative index endoscopic finding initially (11). Therefore, it is presumed that the strategy of screening, surveillance and treatment of esophageal SPN is associated with the prognosis of patients with hypopharyngeal cancer.

The aim of this study was to review the treatment outcomes as well as to understand the impacts of different strategies of endoscopic screening for esophageal SPN on the survival of patients with hypopharyngeal cancer.

## Materials and methods

### Study design

This was a retrospective study that reviewed the medical records of patients with hypopharyngeal cancer from 2008 to 2021. The study protocol was approved by the ethical review committee of Far Eastern Memorial Hospital (IRB No.: 111165-E), and the informed consent form was waived by the review committee. All patients with hypopharyngeal carcinoma who were diagnosed and treated were included for review. Demographic data on age, sex, primary site and TNM stage according to the American Joint Committee on Cancer, AJCC 8th edition of index HNC, treatment method and the pattern of endoscopic examination of the esophagus were reviewed. Whether magnifying endoscopy with narrow-band imaging (ME-NBI) examination of the esophagus was performed or not was recorded, and if patients underwent ME-NBI, they were divided into three groups: (1) ME-NBI within half a year only, (2) ME-NBI performed more than half a year, and (3) ME-NBI within half a year and further regular surveillance. Finally, the overall survival (OS) time of the patients was calculated and defined as the time from diagnosis of hypopharyngeal cancer to the last follow-up time or death time.

### Endoscopic examination of esophageal SPNs

Endoscopic evaluation of the esophagus was performed by gastroscopes with magnifying or near-focus function under the NBI system. Any brownish color change in the esophageal mucosa under NBI was further scrutinized for morphological changes in intraepithelial capillary loops (IPCLs) by magnification. Abnormal mucosa was defined as type B1, B2 and B3 according to the Japanese Esophageal Society classification IPCL by means of ME-NBI examination (12). Biopsies were taken for endoscopically suspicious esophageal neoplasms for histopathological evaluation.

### Statistical analysis

All statistical analyses were performed using STATA, version 14.0. Categorical variables are expressed as numbers (percentages), continuous variables are expressed as the mean values ( ± standard deviation; SD), and follow-up time is expressed as medians (interquartile range; IQR). We used Kaplan–Meier curves to understand the survival situation between different risk factor groups and finally used the log-rank test to compare whether the survival curves of different groups were statistically significant. We further used Cox regression analysis to estimate the impact of various risk factors on survival and calculated hazard ratios (hazard ratios, HRs) and 95% confidence intervals (95% confidence intervals, CIs). A p value <0.05 indicated statistical significance.

## Results

**Figure 1:**
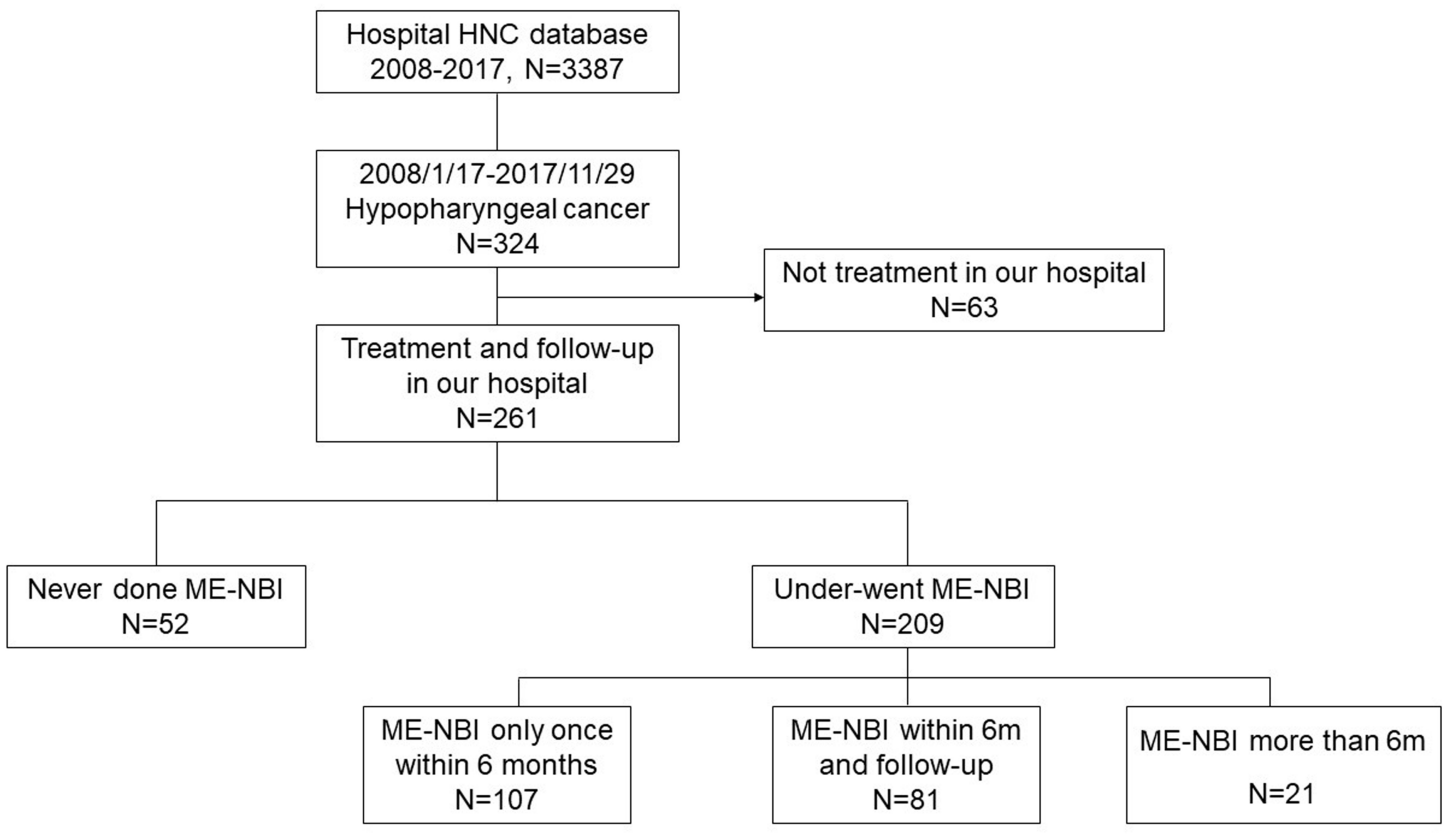
Study flow diagram.

A total of 3,387 patients with HNC were extracted from the database. Among them, 324 patients were diagnosed with hypopharyngeal cancer, and 63 patients were excluded due to a lack of data on the treatment course. Finally, 261 patients with hypopharyngeal cancer were included for analysis (**Figure 1**). Demographic data of the enrolled subjects are shown in **Table 1**. There were 252 (97%) males and 9 (3%) females with a mean age ( ± SD) of 63.2 ( ± 10.4) years. Habits of cigarette smoking, alcohol drinking, and betel nut chewing were as follows: 190 (73%) patients with smoking habits, 21 (8%) without smoking habits and 50 (19%) of unknown; 158 (61%) patients with alcohol drinking habits, 53 (20%) without drinking habits and 50 (19%) of unknown; 123 (47%) patients with betel nut chewing habits, 88 (34%) without chewing habits and 50 (19%) of unknown. For the subsites of index HNC, the pyriform sinus was the most common site with 185 (71%) patients, followed by the posterior pharyngeal wall with 41 (16%) patients and the postcricoid area with 12 (5%) patients. Another 16 (6%) patients had tumors covered on the pyriform sinus and posterior pharyngeal wall, 4 (1%) patients with tumors covered on the pyriform sinus and postcricoid area and 3 (1%) patients with tumors covered on the pyriform sinus and posterior pharyngeal wall and postcricoid area. The distribution of cancer stages was as follows: 21 (8%) patients diagnosed at stage I, 20 (8%) at stage II, 27 (10%) at stage III, 116 (44%) at stage IVA, 65 (25%) at stage IV B and 12 (5%) at stage IVC.

**Table 1:** Demographic data of enrolled subjects with hypopharyngeal cancer (n=261).

Variables		Number (%)/mean ± SD/median (IQR)
**Gender**	Male	252 (97%)
	Female	9 (3%)
**Age**		63.2 ± 10.4 (40-97)
**Cigarette smoking**	Yes	190 (73%)
	None	21 (8%)
	Unknown	50 (19%)
**Alcohol drinking**	Yes	158 (61%)
	None	53 (20%)
	Unknown	50 (19%)
**Betel nuts chewing**	Yes	123 (47%)
	None	88 (34%)
	Unknown	50 (19%)
**Primary site**	Pyriform sinus	185 (71%)
	Posterior pharyngeal wall	41 (16%)
	Post-cricoid area	12 (5%)
	Pyriform sinus、posterior pharyngeal wall	16 (6%)
	Pyriform sinus、post-cricoid area	4 (1%)
	Pyriform sinus、posterior pharyngeal wall、post-cricoid area	3 (1%)
**C-Stage (AJCC 8^th^)**	I	21 (8%)
	II	20 (8%)
	III	27 (10%)
	IVA	116 (44%)
	IVB	65 (25%)
	IVC	12 (5%)
**SPN development**	Yes	67 (26%)
	Synchronous	45 (67%)
	Metachronous	22 (33%)
**SPN site**	Esophagus	50 (75%) (SCC 33 (66%), HGIN 17 (34%))
	Oral cavity	10 (15%)
	Oropharynx	3 (4%)
	NPC	2 (3%)
	Larynx	1 (1%)
	Lung	1 (1%)
**Treatment of primary HNC**	CCRT alone	209 (80%)
	Surgery + CCRT	23 (8%)
	CT alone	14 (5%)
	RT alone	10 (4%)
	Surgery + CT	4 (2%)
	Surgery alone	1 (1%)

AJCC, The American Joint Committee on Cancer; CCRT, concurrent chemoradiotherapy; CT, chemotherapy; EMR, endoscopic mucosal resection; ESD, endoscopic submucosal dissection; HGIN, high-grade intraepithelial neoplasia; HNC, head and neck cancer; IQR, interquartile range; ME-NBI, magnifying endoscopy with narrow-band imaging; NPC, nasopharyngeal cancer; RT, radiotherapy; SD, standard deviation; SPN, second primary neoplasm.

A total of 67 (26%) patients were diagnosed with SPN (**Table 1**), of which 45 (67%) were diagnosed with SPN within half a year and 22 (33%) more than half a year after the diagnosis of the index primary hypopharyngeal cancer. Fifty (75%) patients had SPNs in the esophagus. Among them, 33 (66%) were squamous cell carcinoma (SCC), and 17 (34%) were high-grade intraepithelial neoplasia (HGIN). Other SPN sites were in the oral cavity (10 (15%) patients), oropharynx [3 (4%)], and nasopharynx (2), one with laryngeal cancer and one with lung cancer.

Regarding the location of esophageal SPNs (**Table 2**), the majority (66%) were located in the middle part of the esophagus, whereas 14 (28%) patients had SPNs located in the upper esophagus and 3 (6%) had SPNs located in the lower part. The stages of esophageal cancer were 14 (43%) at stage I (including HGIN), 4 (12%) at stage II, 10 (30%) at stage III and 5 (15%) at stage IV. Among these esophageal cancers, 35 in 50 patients (70%) were synchronous, and the stage distribution was as follows: 13 (36%) patients at stage I, 3 (9%) at stage II, 7 (20%) at stage III, 3 (9%) at stage IV and 9 (26%) with HGIN. Fifteen (30%) patients had metachronous esophageal SPNs, and one (7%) patient was at stage I, one (7%) patient was at stage II, 3 (20%) were at stage III, 2 (13%) were at stage IV and 8 (53%) had HGIN.

**Table 2:** Staging, treatment and surveillance of esophageal SPN.

Variables		Number (%) / mean ± SD/ median (IQR)
**Esophageal SPN location**	Upper	14 (28%)
	Middle	33 (66%)
	Lower	3 (6%)
**Esophageal SCC-T**	1	14 (42%)
	2	6 (18%)
	3	10 (30%)
	4	3 (10%)
**Esophageal SCC-N**	0	20 (61%)
	1	7 (21%)
	2	6 (18%)
**Esophageal SCC-M**	0	32 (97%)
	1	1 (3%)
**Esophageal SPN C-stage**	I	14 (43%)
	II	4 (12%)
	III	10 (30%)
	IV	5 (15%)
**Esophageal SPN Synchronous-T**	**Synchronous**	**35 (70%)**
	1	13 (50%)
	2	5 (19%)
	3	5 (19%)
	4	3 (12%)
**Synchronous-N**	0	19 (73%)
	1	3 (12%)
	2	4 (15%)
**Synchronous-M**	0	25 (96%)
	1	1 (4%)
**C-Stage (AJCC 8th)**	I	13 (36%)
	II	3 (9%)
	III	7 (20%)
	IV	3 (9%)
	HGIN	9 (26%)
**Metachronous-T**	**Metachronous**	**15 (30%)**
	1	1 (14%)
	2	1 (14%)
	3	5 (72%)
	4	0 (0%)
**Metachronous-N**	0	1 (14%)
	1	4 (57%)
	2	2 (29%)
**Metachronous-M**	0	7 (100%)
	1	0 (0%)
**C-Stage (AJCC 8th)**	I	1 (7%)
	II	1 (7%)
	III	3 (20%)
	IV	2 (13%)
	HGIN	8 (53%)
**Treatment of esophageal SPN**	CCRT	23 (46%)
	ESD	13 (26%)
	Follow-up without treatment	4 (8%)
	Surgery	3 (6%)
	CT alone	3 (6%)
	EMR	1 (2%)
	Mortality without treatment	1 (2%)
	RFA	1 (2%)
	APC	1 (2%)
**ME-NBI examination timing**	Never done	52 (20%)
	≤ 6 months of diagnosis of HNC	107 (41%)
	> 6 months of diagnosis of HNC	21 (8%)
	≤ 6 months of diagnosis of HNC and surveillance every 6~12 months	81 (31%)
**Follow-up, years**		1.6 (±2.9) (0.7-3.6)

APC, argon plasma coagulation; AJCC, The American Joint Committee on Cancer; CCRT, concurrent chemoradiotherapy; CT, chemotherapy; EMR, endoscopic mucosal resection; ESD, endoscopic submucosal dissection; RFA, radiofrequency ablation; HGIN, high-grade intraepithelial neoplasia; IQR, interquartile range; ME-NBI, magnifying endoscopy with narrow-band imaging; RT, radiotherapy; SCC, squamous cell carcinoma; SD, standard deviation; SPN, second primary neoplasm.

The treatment strategy for primary HNC included radiotherapy combined with chemotherapy (CCRT) in 209 (80%) patients, followed by surgery with CCRT in 23 (8%) patients, chemotherapy alone in 14 (5%) patients, radiotherapy alone in 10 (4%) patients, surgery and chemotherapy in 4 (2%) patients, and surgery alone in one patient. Surgical procedures included total laryngectomy or partial laryngectomy combined with total or partial pharyngectomy. Regarding the treatment of esophageal SPN, 23 (46%) patients underwent CCRT, 13 (26%) underwent endoscopic submucosal dissection (ESD), 4 (8%) refused treatment, 3 (6%) underwent esophagectomy, 3 (6%) underwent chemotherapy alone, 1 (2%) underwent endoscopic mucosal resection (EMR), 1 (2%) did not undergo treatment due to mortality, 1 (2%) underwent endoscopic radiofrequency ablation (RFA) and 1 (2%) underwent endoscopic argon plasma coagulation (APC).

A total of 209 (80%) patients underwent ME-NBI examination, of which 107 (41%) received ME-NBI screening within half a year after diagnosis, 21 (8%) received ME-NBI screening more than half a year after diagnosis, and 81 (31%) patients received ME-NBI screening within half a year after diagnosis and further regular surveillance. The median (IQR) follow-up period was 1.6 (2.9) years.

**Figure 2:**
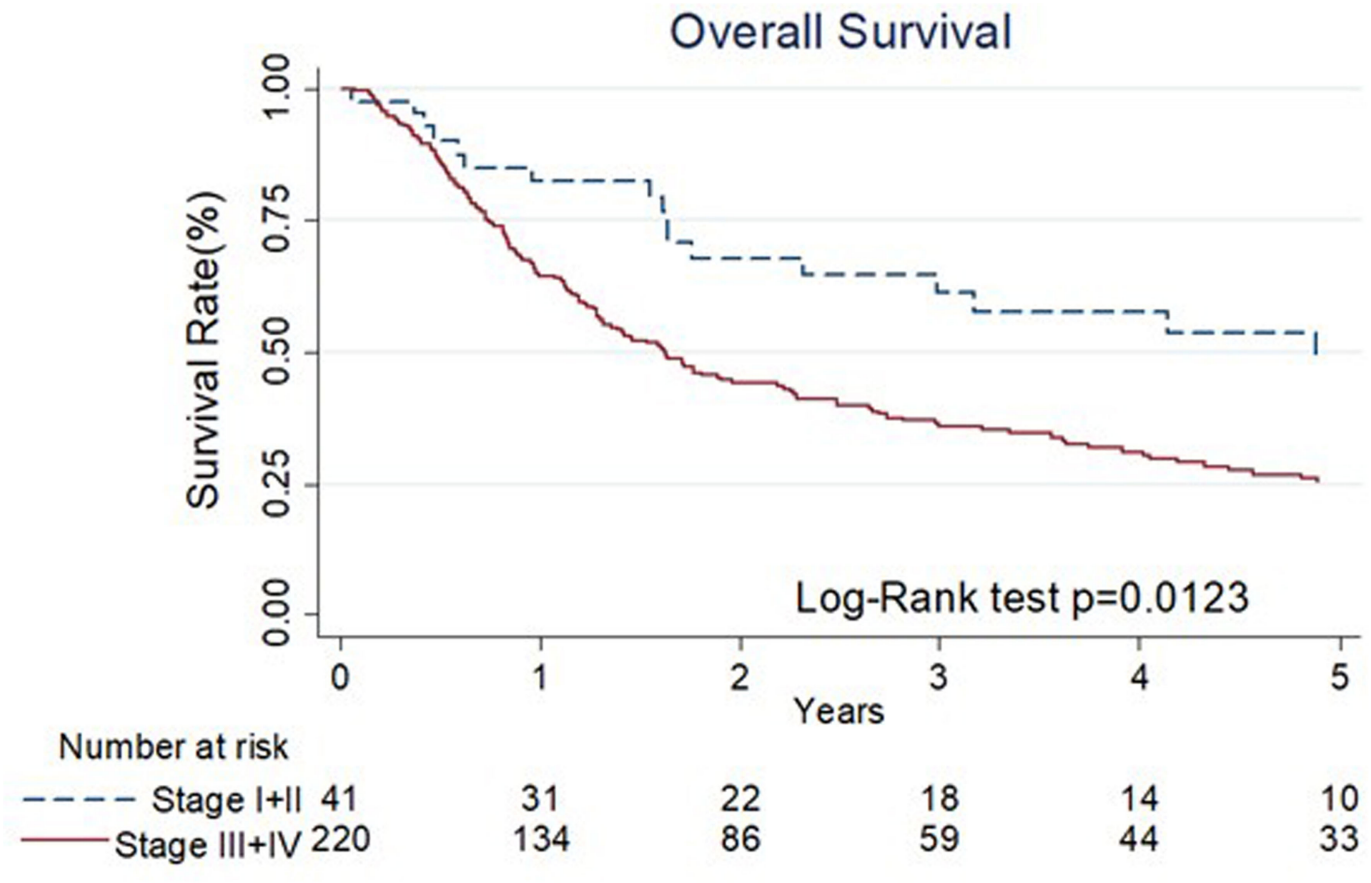
The Kaplan-Meier diagram and log-rank test of overall survival among different stages of primary index cancer.

**Figure 3:**
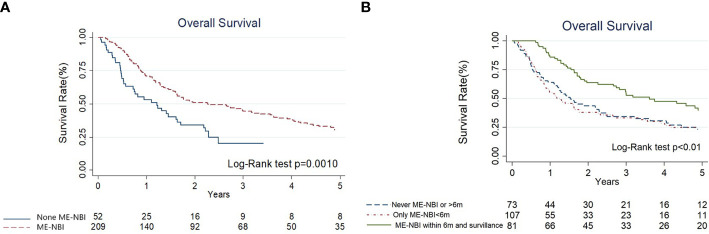
**(A)** The Kaplan-Meier diagram and log-rank test of overall survival among hypopharyngeal cancer patients with and without magnifying endoscopy under narrow-band imaging screening of esophagus. **(B)** The Kaplan-Meier diagram and log-rank test of overall survival according to different endoscopy surveillance strategies.

The results of univariate and multivariate Cox regression analyses of overall survival (OS) are shown in **Table 3**. Cancer stage was associated with OS by univariate analyses (III+IV stage compared with I+II stage, hazard ratio (HR): 1.78, 95% confidence interval (CI) 1.13-2.80, p=0.014). The Kaplan–Meier diagram and log-rank test are shown in **Figure 2**. The univariate Cox regression analysis showed that patients with advanced cancer had poorer survival (p=0.014, **Table 3**, **Figure 2**), while patients who received ME-NBI (p=0.003) showed better OS (**Table 3**, **Figure 3A**). By multivariate analysis, after adjusting for confounders such as sex and age, we found that patients with advanced cancer stage III & IV (compared stage I&II, HR: 1.86, 95% CI 1.18-2.95, p=0.008) had poor survival, and patients who received ME-NBI within half a year and further surveillance follow-up (HR: 0.53, 95% CI 0.36-0.78, p=0.001) had a better prognosis (**Figure 3B**).

**Table 3:** Univariate and multivariate Cox regression analyses of overall survival of enrolled hypopharyngeal cancer patients.

	Univariate		Multivariate	
	HR (95% CI)	p value	HR (95% CI)	p value
Sex				
Female	Ref.		Ref.	
Male	1.05 (0.47-2.38)	0.898	1.33 (0.58-3.04)	0.504
Age				
<65 years old	Ref.		Ref.	
>=65 years old	1.26 (0.94-1.70)	0.118	1.18 (0.88-1.59)	0.275
Stage				
I+II	Ref.		Ref.	
III+IV	1.78 (1.13-2.80)	0.014	1.86 (1.18-2.95)	0.008
Primary site				
Pyriform sinus	Ref.			
Posterior pharyngeal wall	0.81 (0.52-1.25)	0.332		
Post-cricoid area	0.75 (0.35-1.60)	0.454		
Overlapping	1.18 (0.70-1.99)	0.528		
ME-NBI				
No	Ref.			
Yes	0.57 (0.40-0.80)	0.001		
ME-NBI strategy				
Never done or > 6 months	Ref.		Ref.	
Once only ≤ 6 months	1.11 (0.78-1.56)	0.570	1.10 (0.77-1.55)	0.603
≤ 6 months and surveillance	0.55(0.38-0.81)	0.003	0.53 (0.36-0.78)	0.001

HR, hazard ratio; ME-NBI, magnifying endoscopy with narrow-band imaging; Ref., reference.

## Discussion

Our findings are the first to demonstrate improved overall survival in patients with hypopharyngeal cancer undergoing ME-NBI and emphasize the importance of ME screening and further surveillance.

Treatment of hypopharyngeal cancer remains challenging and requires a multidisciplinary team to establish the optimal treatment options. The primary goal is to improve survival from an oncological perspective and provide functional organ preservation wherever feasible (13). Because there is a substantial proportion of patients with HNC with cigarette smoking, chewing betel nuts and drinking alcohol habits, which are common carcinogens for esophageal SCC, the risk of developing malignancies in the entire aerodigestive tract, including the lungs and esophagus, is high (8–10, 14, 15). Several studies have found that a very high proportion of patients with HNC are complicated by SPNs. After comprehensive review and analysis, we found that approximately 12% of HNC patients will develop second primary cancer (9). If the second primary cancer is located in the esophagus, the mortality rate will be higher than those with other SPNs, with a 5-year survival rate of only 6% (9, 10, 14, 15). The synchronous and metachronous rates of the development of esophageal SPNs in HNC patients are approximately 13-23.3% and 10-12%, respectively (7, 11, 16–19). In our study, 70% of esophageal SPNs developed synchronously, while 30% of them were metachronous SPNs (**Table 2**). Fortunately, most of the SPNs of the esophagus detected by screening in patients with HNC are at asymptomatic precancerous dysplastic or early cancer stages (7, 18, 19). According to the results of this study, the esophageal SPNs of HGIN and stage I SCC were 26% and 36% synchronous and 53% and 7% metachronous lesions, respectively (**Table 2**). Therefore, we believe that early diagnosis of esophageal SPN not only provides opportunities for early treatment but also may improve the overall survival rate of patients with HNC (20). Regarding the primary site of index HNC, we found that the risk of hypopharyngeal cancer patients who develop second primary esophageal cancer is four times that of patients with oral cancers (7). Thus, it is presumed that the screening, surveillance and treatment of esophageal SPNs should be an important prognostic strategy for the management of patients with hypopharyngeal cancer.

The survival rate of hypopharyngeal cancer is worse than that of other HNCs. It may be related to concurrent esophageal cancer, nutritional status during treatment, swallowing dysfunction and whether advanced hypopharyngeal cancer patients received aggressive surgical treatment. In patients with HNCs, prognosis may be more affected by the esophageal SPN due to its poorer prognosis as compared to SPN of other sites (14, 15). When estimating the impact of various risk factors on survival among these patients, it is crucial to take into consideration the influence of esophageal cancer. Therefore, screening and surveillance of esophageal SPN, especially at asymptomatic early stages, become of paramount importance to improve overall outcome. In our study, the incidence of HGIN and stage I esophageal SPN were 26% and 36% synchronously, and 53% and 7% metachronously (**Table 2**) which were much higher than those in nationwide data (only 11% at stage I) (21). However, it is not well understood whether routine endoscopic screening of esophageal SPNs and regular follow-up can improve the prognosis of patients with hypopharyngeal cancer. In our previous study of a total of 1,577 HNC patients, those who underwent endoscopic screening of esophageal SPNs with negative findings initially had a better prognosis than those without screening (22). Additionally, with advancements in image-enhanced endoscopy (IEE) technology, particularly NBI systems and chromoendoscopy using iodine-containing solution spraying, the diagnosis of precancerous or early cancerous neoplasia of the esophagus could be achieved (23–25).

**Figure 4:**
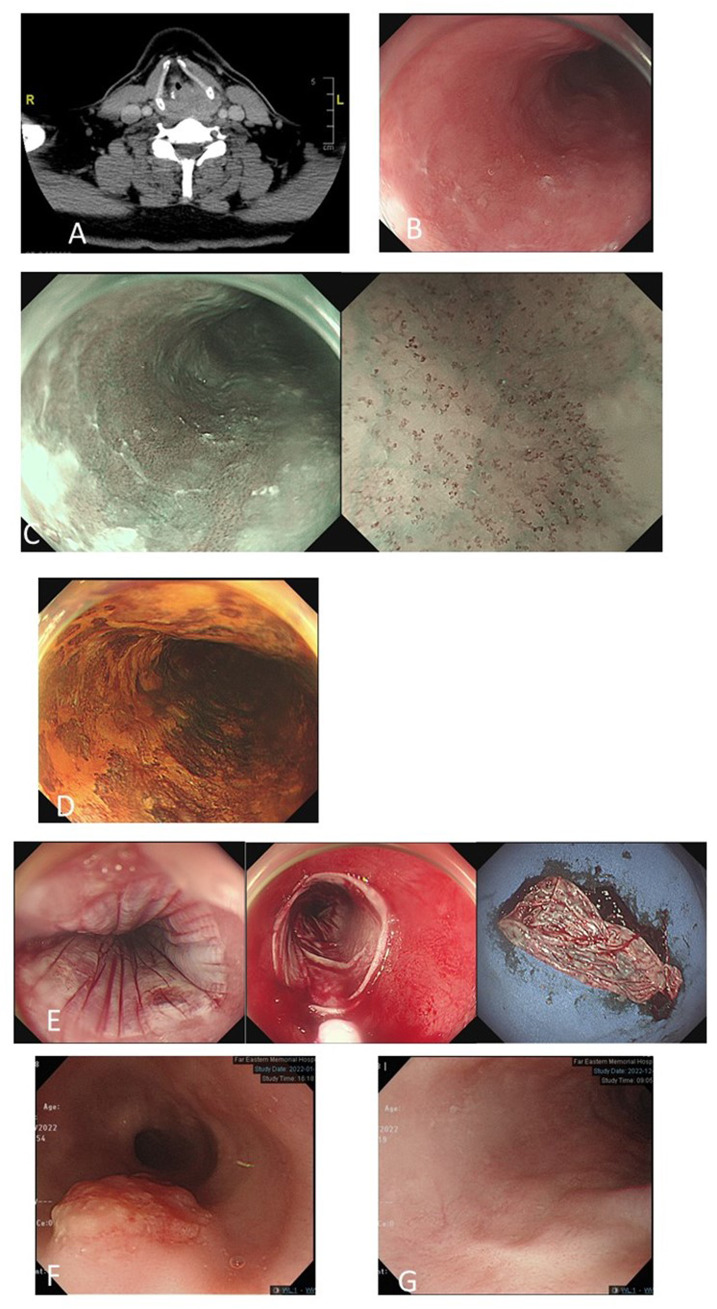
A 52-year-old man with left hypopharyngeal cancer, stage cT4N0M0 **(A)**. Magnifying endoscopy with narrow-band imaging (ME-NBI) endoscopic screening of the esophagus revealed a mild hyperemic surface **(B)** under white-light imaging, brownish discoloration with irregular intraepithelial papillary capillary loops under ME-NBI **(C)** and multifocal Lugol-voiding areas **(D)** in the middle part of the esophagus. Biopsy revealed squamous hyperplasia and low-grade dysplasia. He underwent concurrent chemotherapy followed by total laryngectomy. Follow-up ME-NBI 6 months after completion of the treatment for primary index hypopharyngeal cancer revealed disease progression of dysplastic esophageal mucosa with biopsy reporting high-grade intraepithelial neoplasia. He underwent endoscopic radiofrequency ablation **(E)**. After 4 years of follow-up, an exophytic mass in the upper-middle part of the esophagus **(F)** and biopsy revealed squamous cell carcinoma (cT2N0M0). He received definitive CCRT for esophageal cancer. Six months after the treatment, endoscopy surveillance showed complete resolution of esophageal cancer with scarring **(G)**.

To date, there is no clear consensus or guideline for the treatment of hypopharyngeal cancer with secondary esophageal neoplasia. Early esophageal neoplasia can be managed by minimally invasive endoscopic resection techniques, which provide equivalent long-term survival compared to surgery but better quality of life (26–28). In our study, we aggressively treated primary index hypopharyngeal cancer as well as synchronous or metachronous esophageal SPNs concomitantly. For esophageal SPNs at precancerous or early status, we used endoscopic ablative or resection methods, including EMR, ESD, RFA or APC, according to the conditions of the patients and the characteristics of the lesions (**Table 2**). By proactively managing both primary and second primary neoplasms (**Figure 4**), patients with hypopharyngeal cancer can be maintained in complete remission or stable disease status. In this study, we further categorized the strategy of ME-NBI examination of the esophagus. The results have demonstrated that a better overall survival could be provided to hypopharyngeal cancer patients when ME-NBI screening of synchronous esophageal SPN within 6 months after initial diagnosis of index primary HNC and regular endoscopic surveillance for metachronous lesions can be implemented (**Table 3**, **Figure 3B**).

In our experience, both ME-NBI screening and regular endoscopic surveillance for metachronous lesions are important for the survival of hypopharyngeal cancer patients. In **Figure 4**, we demonstrate one 52-year-old man with left hypopharyngeal cancer, stage cT4N0M0, with initial synchronous low-grade dysplastic esophageal neoplasm after ME-NBI screening. He underwent concurrent chemotherapy followed by total laryngectomy. Surveillance endoscopic examination using ME-NBI 6 months after completion of the treatment of primary index hypopharyngeal cancer revealed disease progression of dysplastic esophageal mucosa, and biopsy reported HGIN. He underwent endoscopic radiofrequency ablation with complete remission. Unfortunately, after 4 years of follow-up, an exophytic mass in the upper-middle part of the esophagus developed, and biopsy revealed squamous cell carcinoma with staging cT2N0M0. He received definitive CCRT for esophageal cancer. Six months after the treatment, endoscopy surveillance showed complete resolution of esophageal cancer with scarring.

There were some limitations of this study. First, it was a retrospective study in a single tertiary hospital, and the results may not be generalized. Second, the timing and surveillance interval of ME-NBI examination of the esophagus was not standardized, and the optimal surveillance intervals could not be revealed. Finally, we did not investigate the cause of death in the survival analysis, and whether patients who died of disease progression of primary index hypopharyngeal cancers or SPN of the esophagus was not well understood.

## Conclusions

In conclusion, we suggest screening esophageal SPNs in all newly diagnosed hypopharyngeal cancer patients as well as regular endoscopic surveillance thereafter. By proactive ME-NBI examination of the esophagus and treatment of primary and secondary neoplasms accordingly, the survival of patients with hypopharyngeal cancer can be improved. We believe that patient adherence to treatment and surveillance program which improves early detection and management of either metachronous primary or secondary tumors and possible lifestyle modification is one of the direct impacts on cancer outcome.

## Data availability statement

The original contributions presented in the study are included in the article/Supplementary Material. Further inquiries can be directed to the corresponding author.

## Ethics statement

The studies involving humans were approved by ethical review committee of Far Eastern Memorial Hospital (IRB No.: 111165-E). The studies were conducted in accordance with the local legislation and institutional requirements. Written informed consent for participation in this study was provided by the participants’ legal guardians/next of kin.

## Author contributions

Study conception and design: C-SC, C-YW, Y-HL, W-CL, P-CC, W-LH, and L-JL. Acquisition of data: C-SC, C-YW, YHL, and L-JL. Analysis and interpretation of data: C-SC, C-YW, W-CL, P-CC, W-LH, and L-JL. Drafting of manuscript: C-SC, C-YW, and L-JL. Critical revision: C-SC, C-YW, and L-JL. All authors contributed to the article and approved the submitted version.
